# Use of organic fertilizers in solar photo-Fenton process as potential technology to remove pineapple processing wastewater in Costa Rica

**DOI:** 10.12688/openreseurope.14997.1

**Published:** 2022-09-01

**Authors:** Dayatri Vanessa Bolaños Picado, Mario Masis Mora, Esteban Duran Herrera, Luís Fernando Pérez Mercado, Núria López Vinent, Alberto Cruz Alcalde, María Mercedes Alvarez Caero, Carlos Esteban Rodríguez Rodríguez, Carmen Sans Mazón

**Affiliations:** 1 Escuela de Ingeniería Química, Universidad de Costa Rica, San José de Costa Rica, 11501-2060, Costa Rica; 2 Centro de Investigación en Contaminación Ambiental, Universidad de Costa Rica, San José de Costa Rica, 11502, Costa Rica; 3 Centro de Aguas y Saneamiento Ambiental, Universidad Mayor de San Simón de Cochabamba, Cochabamba, JV44+W59, Bolivia; 4 Department of Chemical Engineering and Analytical Chemistry, Faculty of Chemistry, Universitat de Barcelona, Barcelona, 08028, Spain

**Keywords:** Organic fertilizers, Pineapple wastewater, Solar-based process, Wastewater treatment

## Abstract

**Background: **This work studied the use of the organic fertilizers DTPA-Fe and EDDS-Fe as iron chelates for solar driven photo-Fenton process at natural pH. This process was proposed to investigate its performance on removing a mixture of agrochemicals (propiconazole, imidacloprid and diuron) from pineapple processing wastewater to obtain a suitable effluent to be reused in the agricultural sector.

**Methods: **Experiments were carried out in a solar simulator with a stirred cylindric photoreactor, with a volume of 150 mL and controlled temperature (20°C). The first set of experiments was carried out with ultrapure water to determine optimal iron and H _2_O _2_ concentrations. The second was performed with simulated wastewater of pineapple processing.

**Results: **The optimized operational conditions for both iron complexes were 10 mg L ^-1^ of Fe (III) and 25 mg L ^-1^ of H _2_O _2_, since more than 80% of micropollutants (MP) (at an initial concentration of 1 mg L ^-1^ of each compound) were removed in only 20 min with both DTPA-Fe and EDDS-Fe. The effect of organic matter and inorganic salts on radicals scavenging and chelates stability was also investigated in the experiments performed with synthetic pineapple processing wastewater. The results disclosed differences depending on the iron complex. Nitrites were the principal component influencing the tests carried out with EDDS-Fe. While carbonates at low concentration only significantly affected the experiments performed with DTPA-Fe, they were the major influence on the MPs removal efficiency decrease. In contrast, the presence of Ca ^2+ ^and Mg ^2+^ only influence on this last one. Finally, the results of phytotoxicity disclosed the suitability of treated effluent to be reused in the agricultural sector.

**Conclusions: **This work demonstrated that solar powered photo-Fenton catalysed by iron fertilizer EDDS is a suitable technology for depolluting water streams coming from pineapple processing plants at circumneutral pH, and its subsequent reuse for crop irrigation.

## Plain language summary

Some of the main water uses and waste generation points associated with the pineapple industry include the washing steps for raw produce. Wastewater characteristics are the wide wastewater volume and the significant concentration of agrochemicals. Solar-driven photo-Fenton process is considered one of the most effective treatments for the removal of organic contaminants, like pesticides, from wastewaters. However, the low pH required to support the Fenton reaction (optimum pH of 2.8) represents an important limitation for its application to different nearly neutral effluents. To overcome the narrow pH operation range, chelating agents have been employed to keep iron complexed, thus preventing its precipitation. Several of these organic chelating agents are approved by the European Commission (2003) to be used as fertilizers. This work studied the use of the organic fertilizers DTPA-Fe and EDDS-Fe as iron chelates for solar-driven photo-Fenton process at natural pH. This process was proposed to investigate its performance on removing a mixture of agrochemicals (propiconazole, imidacloprid and diuron) from simulated pineapple processing wastewater to obtain an effluent suitable for reuse in the agricultural sector.

## Introduction

Worldwide agricultural production is dependent on the use of agrochemicals to support human population needs ( Carvalho, 2006). Despite the benefits derived from the use of pesticides at agricultural level, several adverse consequences, mostly related to environmental contamination, are linked to this practice ( Aktar *et al.*, 2009).

Pineapple represents a major crop in Costa Rica; its production covers an area of 58,442 Ha (by 2015), mostly distributed in the lowlands of the northern and Caribbean regions, as well as in the south Pacific coast of the country ( Brenes-Alfaro *et al.*, 2021; MINAE, 2015). Moreover, Costa Rica is the largest exporter of fresh pineapple worldwide, with a production estimated to account for 1.7% of the GDP of the country ( Chen *et al.*, 2020). However, concern has been raised around the impacts of pineapple production, particularly regarding the large amount of plant waste produced and the hazard due to pesticide use ( Chen *et al.*, 2020; Echeverría-Sáenz *et al.*, 2012). Recent monitoring (2015–2018) of surface water and groundwater in areas of pineapple production influence in Costa Rica revealed the occurrence of 28 different pesticides, including herbicides (ametryn, bromacil, diuron, hexazinone, prometryn, paraquat), fungicides (carbendazim, metalaxyl, myclobutanil, paclobutrazol, procloraz, propiconazole, thiabendazole, triadimefon, triadimenol), and insecticides (carbofuran, carbaryl, cyromazine, chlorpyrifos, dichlorvos, diazinon, dimethoate, ethoprophos, imidacloprid, malathion, methoxyfenozide, oxamyl, imazalil, imidacloprid) (unpublished data, Research Center of Environmental Contamination, CICA, Universidad de Costa Rica); of these agrochemicals, 16 are indeed approved for application on pineapple crops, while for instance, bromacil was completely banned in Costa Rica in 2017 (decreet N° 40423-MAG-MINAE-S; La Gaceta, 2017) due to its systematic detection during the monitoring, particularly in groundwater.

Among these pesticides, diuron, imidacloprid and propiconazole have been frequently detected in the monitoring; moreover, diuron and propiconazole were also found in sediments, while imidacloprid was also detected in groundwater (unpublished data, Research Center of Environmental Contamination, CICA, Universidad de Costa Rica). These three pesticides are also simultaneously approved for use in other crops, including banana and plantain, not only in Costa Rica ( SFE, 2022) but also at other latitudes, such as in Bolivia ( Bickel, 2018). Diuron is a phenylurea pre-emergence herbicide, considered as persistent in soil due to its high DT50 of 147–229 d ( Lewis *et al.*, 2016). Its ecotoxicity is considered as moderate to typical taxa (birds, fish, daphnids, bees and earthworms), both at acute and chronic levels; specific effects on non-target communities are reviewed by Giacomazzi and Cochet (2004). Imidacloprid is a neonicotinoid insecticide, known to adversely affect pollinator communities; for this reason, it was banned in the European Union in 2018 for use in crops pollinated by bees ( Declan, 2018); nonetheless, this compound is still extensively used elsewhere. It is a persistent compound, with a soil DT50 of 174–91 d; besides, its acute contact and oral toxicity towards bees, and ecotoxicity towards birds is also described as high ( Lewis *et al.*, 2016). On the other hand, propiconazole is a triazole fungicide; as most triazoles, this is a moderate to persistent pesticide, with a soil DT50 of 35–72 d ( Lewis *et al.*, 2016). High ecotoxicity of this compound has been described at chronic level in fish.

Advanced oxidation processes (AOPs) are regarded as attractive options for the treatment of on-farm pesticide-containing wastewater, as most organic micropollutants (MPs) are prone to attack by hydroxyl radicals (HO•). Among AOPs, the Fenton, and in particular the photo-Fenton process, has been described as effective for the removal of diverse pesticides, including azoxystrobin, bentazone, chlortoluron, carbofuran, cyprodinil, diazinon, dimethoate, endosulfan, formetanate, fludioxonil, hexaconazole, imidacloprid, kresoxim-methyl, luferunon, methamidophos, methomyl, oxamyl, pirimicarb, propamocarb, propyzamide pyrimethanil, tebuconazole and triadimenol, among others ( Abdessalem *et al.*, 2010; Fallmann *et al.*, 1999; Navarro *et al.*, 2011; Tamimi *et al.*, 2008; Zekkaoui *et al.*, 2021). Nonetheless, the low pH required to support the Fenton reaction (optimum pH of 2.8) represents an important limitation for its application to different nearly-neutral effluents; this drawback, along with the significant iron sludge-related secondary pollution, decreases the appeal of the process for full-scale application ( Oller & Malato, 2021). To overcome the narrow pH operation range, and work at the pre-existent pH of polluted effluents (near neutrality in many cases), chelating agents have been employed in order to keep iron complexed, thus preventing its precipitation at a wider pH range ( Gonçalves *et al.*, 2020). Among these chelating agents, polycarboxylates such as citrate and oxalate, and aminopolycarboxylic acids such as EDTA (ethylenediaminetetraacetic acid), and more recently EDDS (ethylenediamine- *N,N’*-disuccinic acid), EDDHA (ethylenediamine- *N,N′*-bis(2-hydroxyphenylacetic acid)) and DTPA (diethylene triamine pentaacetic acid) have been employed in photo-Fenton processes at non-acidic conditions for the removal of micropollutants, including pharmaceuticals or pesticides ( Gonçalves *et al.*, 2020; López-Vinent *et al.*, 2021a; Lumbaque *et al.*, 2019; Papoutsakis *et al.*, 2015; Soriano-Molina *et al.*, 2018) or even for microbial inactivation ( Nahim-Granados *et al.*, 2019). 

Several of these organic chelating agents are approved by the European Commission (2003) to be used as fertilizers, as their-ferric chelates can be applied as iron sources to crops for enzymatic and chlorophyl production purposes. Therefore, their use in the treatment of pesticide-containing wastewater of agricultural origin could result in the production of safe treated water for irrigation with no need for chelate separation ( López-Vinent *et al.*, 2020). Among these compounds, EDDS is a structural isomer of EDTA; however, as it exhibits higher biodegradability, it is considered as an environmentally safe alternative to EDTA ( Wu *et al.*, 2014). Due to its low stability constant with iron, its use in photo-Fenton reactions is expected to result in high initial removal rates, but also, adversely, in rapid iron precipitation which might hinder its global efficiency ( López-Vinent *et al.*, 2021a). Conversely, DTPA, another authorized fertilizer, is known to produce highly stable DTPA-iron complexes compared to other organic chelating agents (including EDDS), which translates into slower removal rates, but also in lower residual iron precipitation after treatment ( López-Vinent *et al.*, 2020). 

Hence, the goal of this work was to compare the effect of two chelating agents used as fertilizers, DTPA and EDDS, on the simultaneous removal of diuron, imidacloprid and propiconazole from synthetic wastewater, by a solar-driven photo-Fenton process, as an eco-friendly solution to produce reusable water for on-farm irrigation. The proposed strategy represents a green option treatment, given that: i. it relies on solar light and the use of hydrogen peroxide, a cheap and “clean” reagent that is transformed into water and molecular oxygen; ii. it permits the treatment of pesticide-containing effluents at normal near-neutral pH, that is, the acidification of a regular homogeneous Fenton is not required; and iii. the residual chelating agents have fertilizer properties and may enhance farm production after treated water is reused in irrigation.

## Methods

### Chemicals

Imidacloprid (IMID), diuron (DIU), propiconazole (PROP), EDDS-Na solution and catalase from bovine liver were acquired from Sigma-Aldrich. Iron chloride (FeCl _3_·6H _2_O), hydrogen peroxide (H _2_O _2_, 30% w/v), acetonitrile and ortoposphoric acid were purcheased from Panreac Quimica. DTPA-Fe (containing 7% of iron) was bought from Phygenera, (Germany).

### Water samples

The first set of experiments was carried out with ultrapure water to determine the effective concentrations of iron and H _2_O _2_. The second one was performed with synthetic wastewater of pineapple processing water. The constituents of this water were: CaCl _2_ (7.7 mg L ^-1^); MgSO _4_ (4.8 mg L ^-1^); FeSO _4_ (1.0 mg L ^-1^); NaNO _2_ (25.8 mg L ^-1^); Na _2_CO _3_ (30.2 mg L ^-1^); Humic acids (2.0 mg L ^-1^).

### Experimental procedure

**Figure 1.**
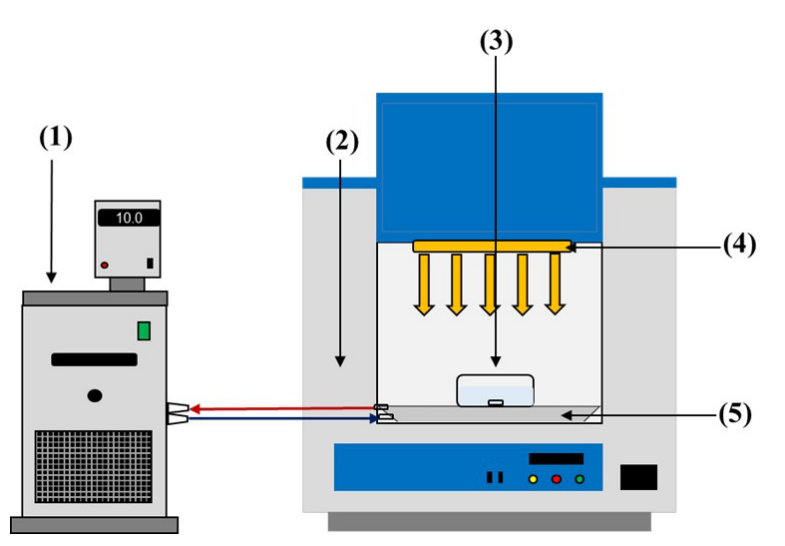
Experimental setup. (1) Thermostatic bath; (2) Solar simulator chamber; (3) Photoreactor; (4) Xenon lamp; (5) Magnetic stirrer.

All experiments were carried out in a solar simulator (Xenonterm-1500RF.CCI) equipped with a Xenon lamp (1.5 kW) (wavelength range: 290–400 nm). The average intensity of incident light was measured with a spectrometer StellarNet Blue-Wave and set to 10 W m ^-2^ for all the experiments. The cylindric photoreactor (4.5 cm height × 9 cm diameter) was located on a magnetic stirrer in the solar simulator. The total volume of each experiment was 150 mL, and the tests were performed at controlled temperature in the solar simulator chamber (20°C). The evaporation was measured during the entire experiment resulting in less than 1% of the total volume. More information about the experimental set-up can be found in Figure 1.

To prepare the dissolution with the iron chelate DTPA-Fe, an appropriate amount of it was added to Milli-Q water. The concentrations were calculated according to the percentage of iron content (7%) in order to obtain concentrations of 2.5, 5 and 10 mg L ^-1^ of iron in solution. The molar ratio of DTPA:Fe was 1.5:1. In the mixture with EDDS, which was not acquired as iron chelate, the same molar ratio than DTPA:Fe was selected. In this case, the EDDS was firstly added to the solution and then the iron, to ensure a good chelation and avoid iron precipitation. The obtained solution was then added in appropriate amounts to Milli-Q water in order to obtain the defined iron concentrations ( *i.e.* 2.5, 5, 10 mg L ^-1^). IMID, DIU and PROP were spiked to the solution to obtain a concentration of 1 mg L ^-1^ of each compound (totalizing 3 mg L ^-1^). Finally, hydrogen peroxide (50 mg L ^-1^) was added in appropriate concentrations (15, 25 and 50 mg L ^-1^) in order to begin the reaction. Samples of 1 mL were retired periodically from the reactor during 60 min and liver bovine catalase was employed to stop the reaction (10 μL of liver bovine catalase at a concentration of 200 mg L ^-1^ to 1 mL of each sample). The samples to analyze the total iron content ( *i.e.* one at the beginning and one at the end of the reaction) were filtered with 0.22 μm PVDF filter to ensure a good read of soluble (chelated and not) iron. Finally, ascorbic acid was added to the sample to have the total soluble iron.

### Analytical techniques

The concentration of the three pesticides (IMID, DIU and PROP) was determined by high performance liquid chromatography (HPLC Infinity Series, Agilent Technologies), using a C-18 Tecknokroma column (250 × 4.6 mm i. d; 5 μm particle size). The HPLC mobile phases A and D were water acidified with orthophosphoric acid (pH = 3) and acetonitrile, respectively. The mobile phase eluent gradient started with 45% eluent D for 5 min, followed by a 1-min linear gradient to 60% D. This condition (60% D) was kept for 16 min, followed by a 1-min gradient back to 45% D, maintained for 5 min. The flow rate was 1 mL min ^-1^ and the injection volume was set to 100 μL. Three wavelengths were fixed according to the absorbance of each compound: 200, 247 and 270 nm for PROP, DIU and IMID, respectively. The monitoring of H _2_O _2_ and total iron in solution were performed by colorimetric method of metavanadate ( Pupo Nogueira *et al.*, 2005) and o-phenanthroline procedure (ISO 6332), respectively. To determine the phytotoxicity, the methodology proposed by Tam and Tiquia ( Tam & Tiquia, 1994) was followed using seeds of *Eruca sativa*.

## Results and discussion

### Determination of effective H _2_O _2_ and Fe(III) concentrations

**Figure 2.**
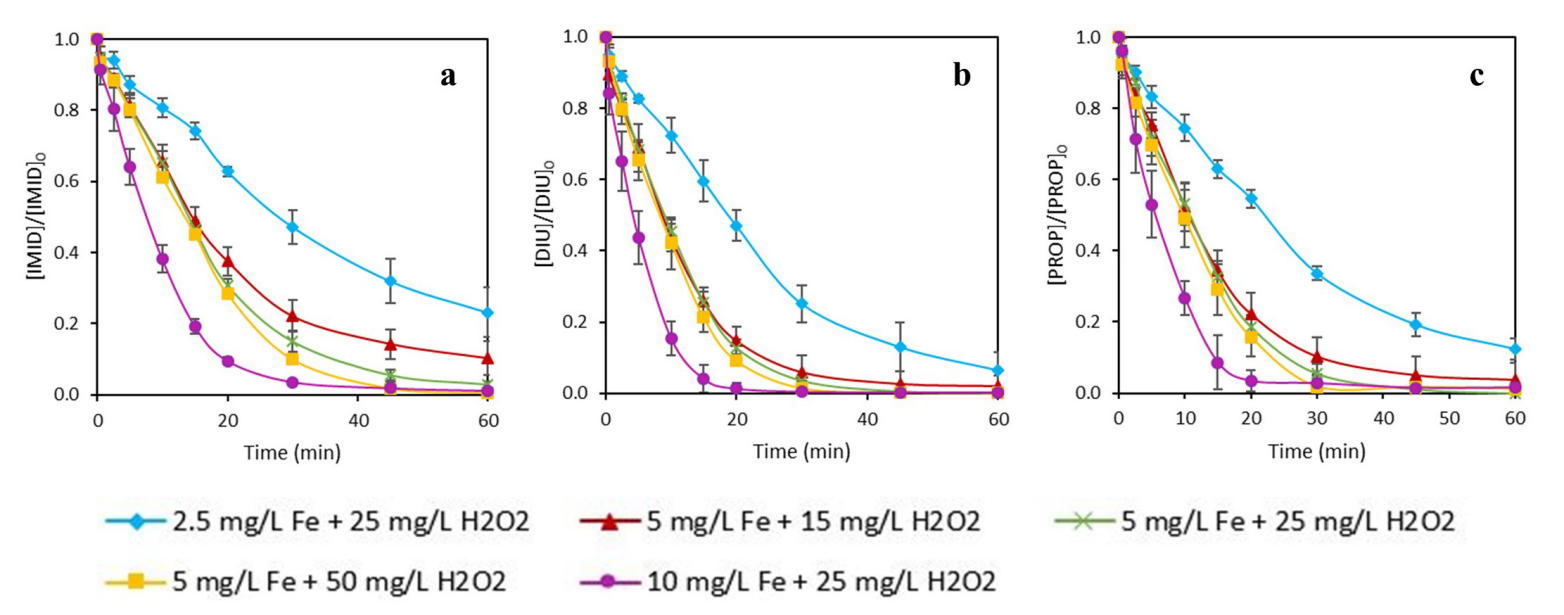
Simultaneous removal of **a**) imidacloprid (IMID), **b**) diuron (DIU) and **c**) propiconazole (PROP) by photo-Fenton process with different initial concentrations of Fe (III) and H _2_O _2_ using DTPA as an iron chelate. [IMID] _0_=[DIU] _0_=[PROPI] _0_=1 mg L ^-1^.

**Figure 3.**
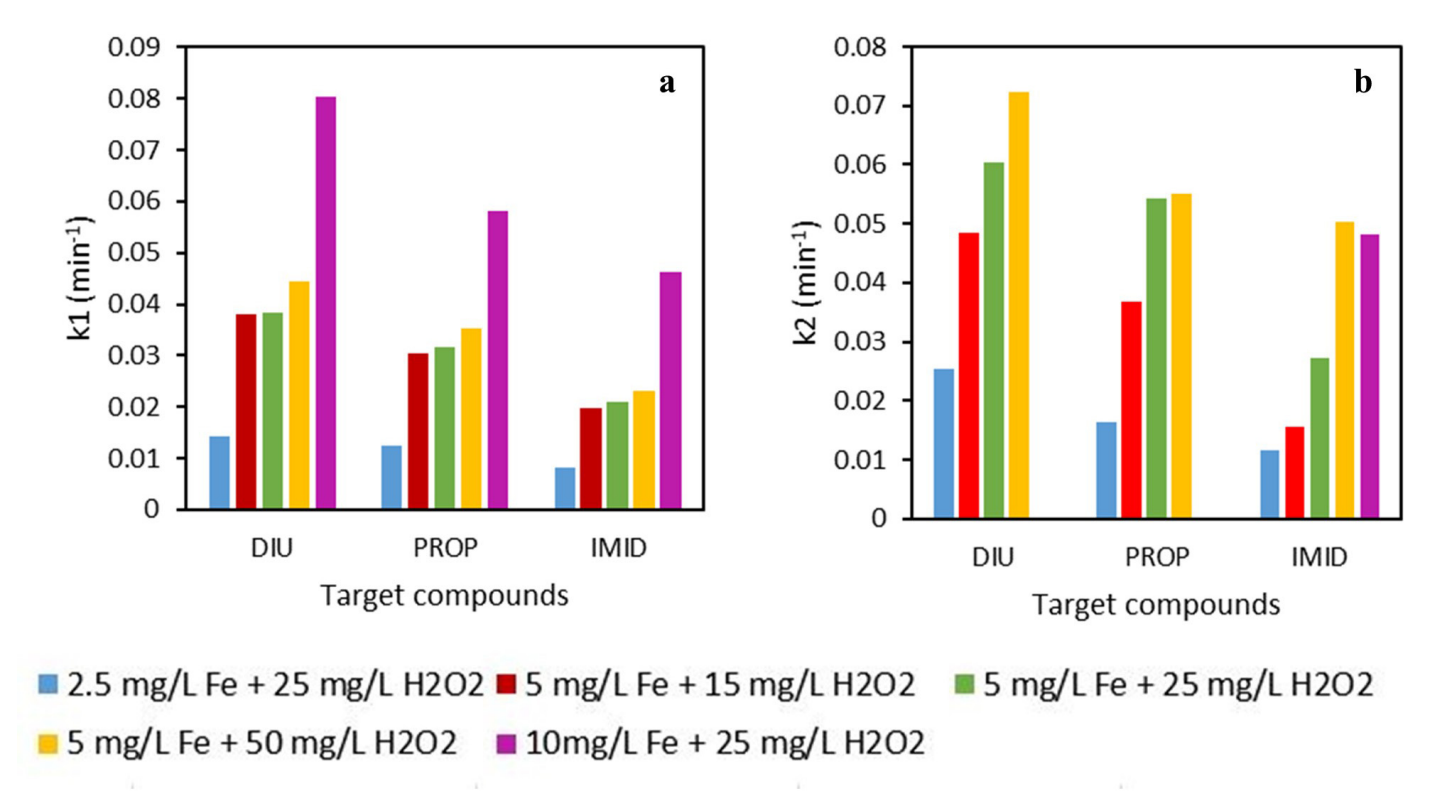
Pseudo-first order kinetic of different experimental conditions using DTPA-Fe iron complex. **a**) k _1_ is the kinetic constant at initial times (0–15 min) and **b**) k _2_ is the kinetic from 15–60 min.

**Figure 4.**
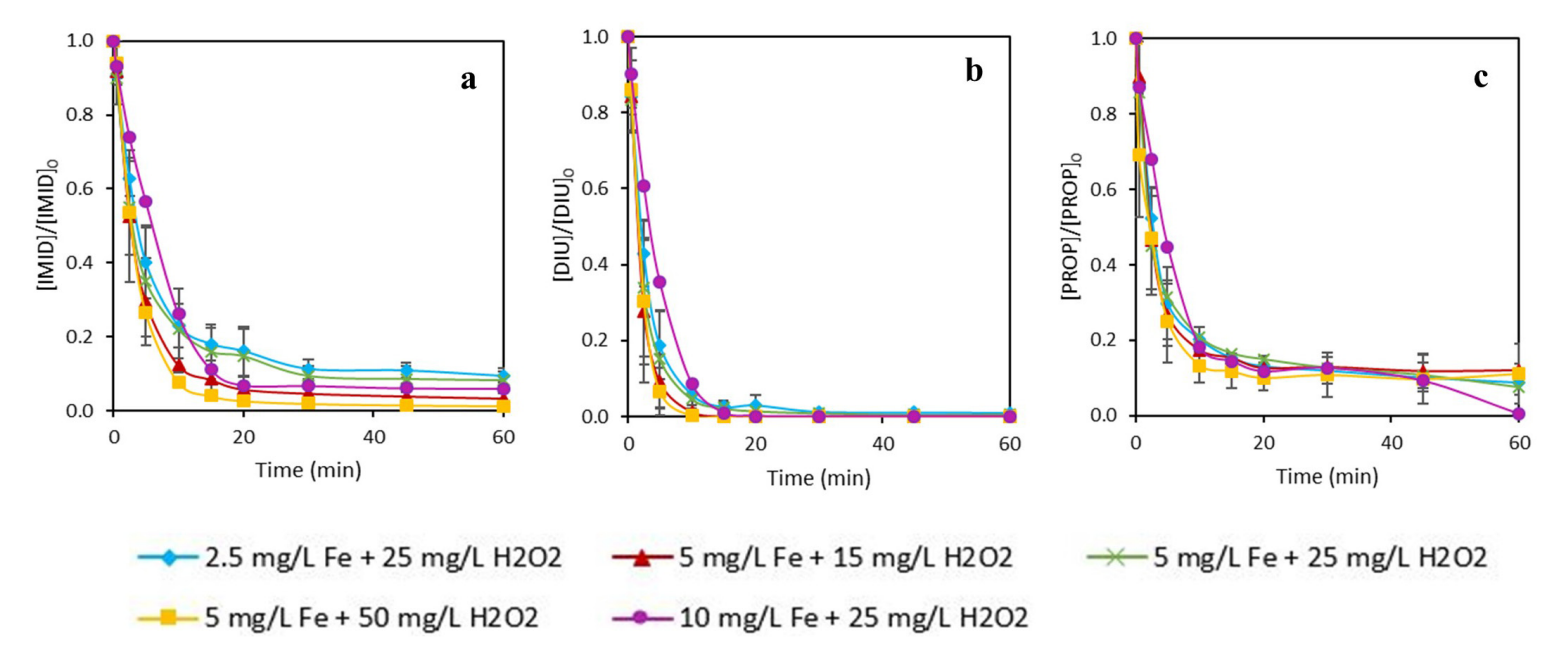
Simultaneous removal of **a**) imidacloprid (IMID), **b**) diuron (DIU) and **c**) propiconazole (PROP) by photo-Fenton process with different initial concentrations of Fe (III) and H _2_O _2_ using EDDS as an iron chelate. [IMID] _0_=[DIU] _0_=[PROPI] _0_= 1 mg L ^-1^.

**Figure 5.**
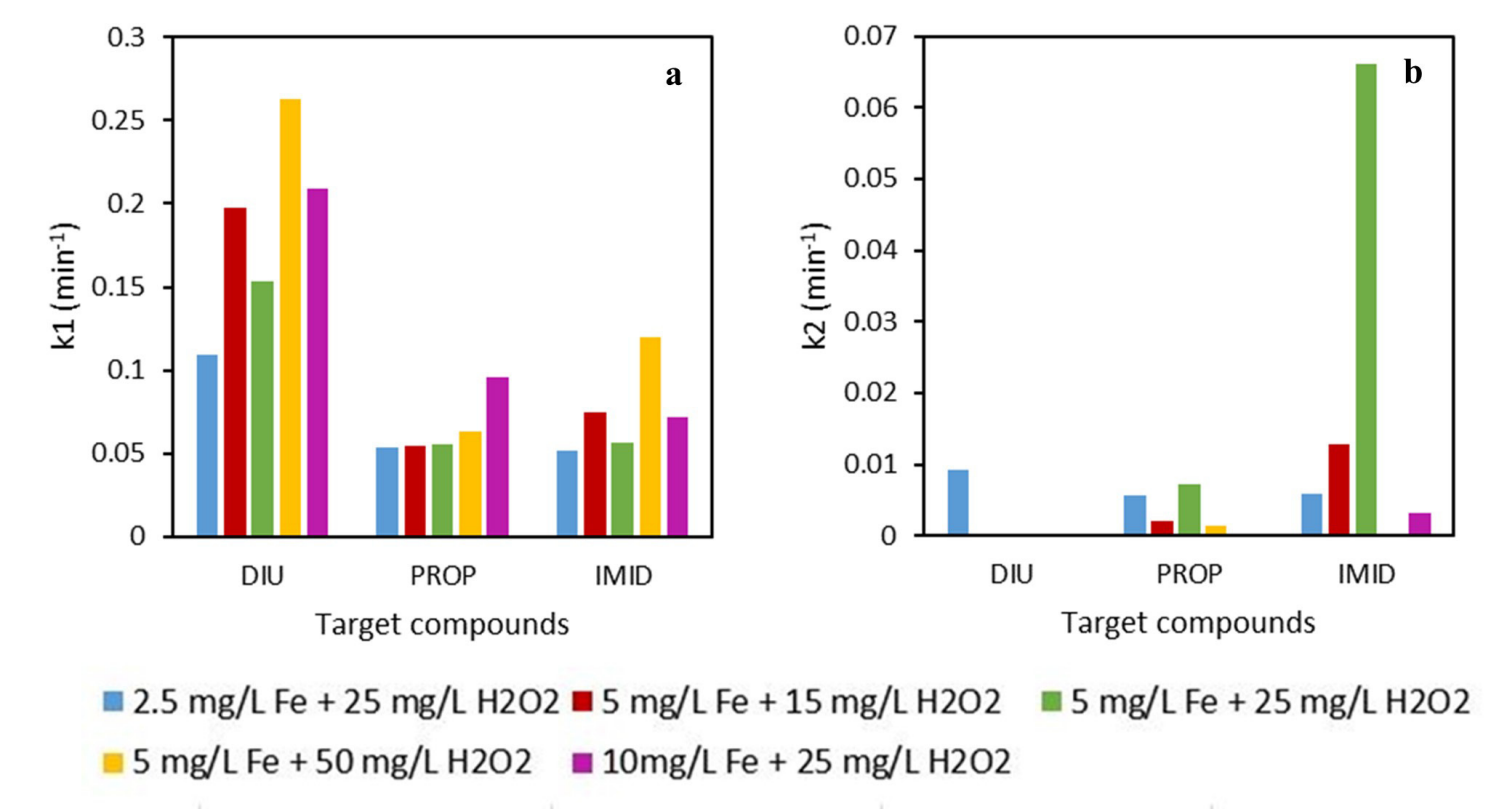
Pseudo-first order kinetic of different experimental conditions using EDDS-Fe iron complex. **a**) k _1_ is the kinetic constant at initial times (0–15 min) and **b**) k _2_ is the kinetic from 15–60 min.

The optimal conditions for simultaneous abatement of the three target micropollutants (IMID, DIU and PROP) by neutral photo-Fenton process was studied by performing such a process at different initial concentrations of Fe and H _2_O _2_, and with two Fe-chelating agents (DTPA and EDDS). The pH of the solution was between 5.5–6.5 during the entire experiment and the tests were carried out in ultrapure water. The results are shown in Figure 2 and Figure 4, for DTPA and EDDS, respectively. Additionally, Figure 3 and Figure 5 gather the observed pseudo-first order kinetics for each experiment. The photolysis was also studied for the three micropollutants. The removal results of that test at the end of the experiment (60 min) were: 4.8, 5.1 and 6.1% for IMID, DIU and PROP, respectively. All experiments were performed in triplicate.

From the results displayed in Figure 2 a-c, it can be observed that the worst removals were observed with the lowest Fe concentration (2.5 mg L ^-1^). With this test, total MP removal at the end of the treatment was not achieved. The best removal rate was seen on DIU, of which about 90% were removed at 60 min. The best MP degradations for three MPs were achieved by the highest iron dose (10 mg L ^-1^). For instance, DIU was degraded 99% in only 20 min. These two experiments were performed with the same H _2_O _2_ concentration, so, the results revealed the importance of the iron concentration to achieve fast kinetics of MPs removal. For example, in the case of DIU, the removal kinetic (k1, see Figure 3a) was 5.7 times higher using 10 than 2.5 mg L ^-1^ of iron.

Regarding the experiments performed with the same concentration of iron (5 mg L ^-1^) but different H _2_O _2_ concentrations, it could be observed in Figure 2 a–c, that the best condition was with 50 mg L ^-1^ of H _2_O _2_, which was the highest one. Nevertheless, the differences in MPs removal between the three experiments were lower than the tests aforementioned (same H _2_O _2_, different iron concentration). The greatest difference was observed in the removal of IMID. About 90% of IMID degradation was obtained at 60 min for the conditions with low H _2_O _2_ concentration (5 mg L ^-1^ of Fe and 15 mg L ^-1^ of H _2_O _2_), against 99% with high H _2_O _2_ dose (5 mg L ^-1^ of Fe and 50 mg L ^-1^ of H _2_O _2_); however, IMID degraded just 1.2 times faster (see Figure 3a, k1) than in the experiment with the lowest H _2_O _2_ concentration. These results evidenced again the importance of the iron concentration in the photo-Fenton experiments, since by increasing the H _2_O _2_ concentration 3.3 times, the kinetic rate only was 1.2 times higher. However, increasing the iron concentration four times, the kinetic rate increased 5.7 times. The results are in accordance with the study performed by López-Vinent and co-authors ( López-Vinent *et al.*, 2019). In that work, by increasing the iron concentration four times, total MP removal was achieved at 20 min while only 60% removal was obtained with the low iron concentration. Nevertheless, with the same iron dose but increasing the H _2_O _2_ concentration six times, similar removal rates were seen at the end of the treatment.

Concerning the differences in MP removal, it can be observed that IMID presented the lowest removal rates in all conditions. The best degradations were achieved for DIU. For instance, observing the experiment ( Figure 2a-c) performed with 5 mg L ^-1^ of Fe and 15 mg L ^-1^ of H _2_O _2_, it could be noted that at 30 min 78% of IMID was eliminated, while 94% and 90% was obtained for DIU and PROP. The results obtained for DIU are in accordance with its kinetic constant with hydroxyl radical (4.75 × 10 ^9^ M ^-1^ s ^-1^) ( Oturan *et al.*, 2011), which is the highest one. Nevertheless, PROP presents the lowest kinetic constant (3.3 × 10 ^9^ M ^-1^ s ^-1^) ( Hong *et al.*, 2022) but the removal was similar to DIU. That fact could evidence the potential generation of other reactive oxygen species (ROS) apart from the formation of hydroxyl radicals, since the photolysis was similar for three MPs. On the other hand, IMID had a kinetic constant of 4.30 x 10 ^9^ M ^-1^ s ^-1^ ( Zaror *et al.*, 2010) but, as aforementioned, the lowest removals.

All these experiments were also performed using EDDS as an iron chelate. From several investigations it was revealed the differences on MPs removal using different iron complexes like EDDS, DTPA, HEDTA, EDDHA ( López-Vinent *et al.*, 2021a; Nahim-Granados *et al.*, 2019). From the study of López-Vinent and coworkers ( López-Vinent *et al.*, 2021a) it was revealed that the removal kinetics are linked to the stability of the chelating agent with iron. For this reason, in this work two iron chelates were tested since they present different stability with iron (k _stab_ DTPA-Fe(III)= 28.60 and k _stab_ EDDS-Fe(III)= 22.0) ( López-Vinent *et al.*, 2021a) The results are displayed in Figure 3a-c.

As can be observed in Figure 4a-c, a similar trend was seen using EDDS and DTPA for experiments using different iron concentrations but equal H _2_O _2_ doses. Observing Figure 3a (which corresponds to the removal of IMID), since it is the one that presents the highest differences between the experiments, the test using the lowest iron concentration (2.5 mg L ^-1^) achieved lower IMID removal than the experiment using 10 mg L ^-1^ of Fe. However, in that case the kinetic (see Figure 5a, k1) was only 1.4 times higher with 10 mg L ^-1^ than 2.5 mg L ^-1^ of Fe. Thus, the improvement on MP removal with increased iron concentration was lower using EDDS than DTPA, with the increment being 5.7 times higher with 10 mg L ^-1^ than 2.5 mg L ^-1^ of iron.

In general, the differences between experimental conditions with EDDS were very low, being practically equal in the removal of PROP and DIU. This fact could be related to the high degradation kinetics during the first 15 min of the reaction. Curiously, after these 15 min, a plateau of the curve was observed in the removal of IMID and PROP. In almost all cases, total DIU was removed at 15 min. The plateauing degradation could be related to the high consumption of H _2_O _2_ in the first 20 min. Between 50–80% of the total H _2_O _2_ was consumed at that time, leading to a limiting step in the oxidation and decreasing the generation of hydroxyl radicals.

**Figure 6.**
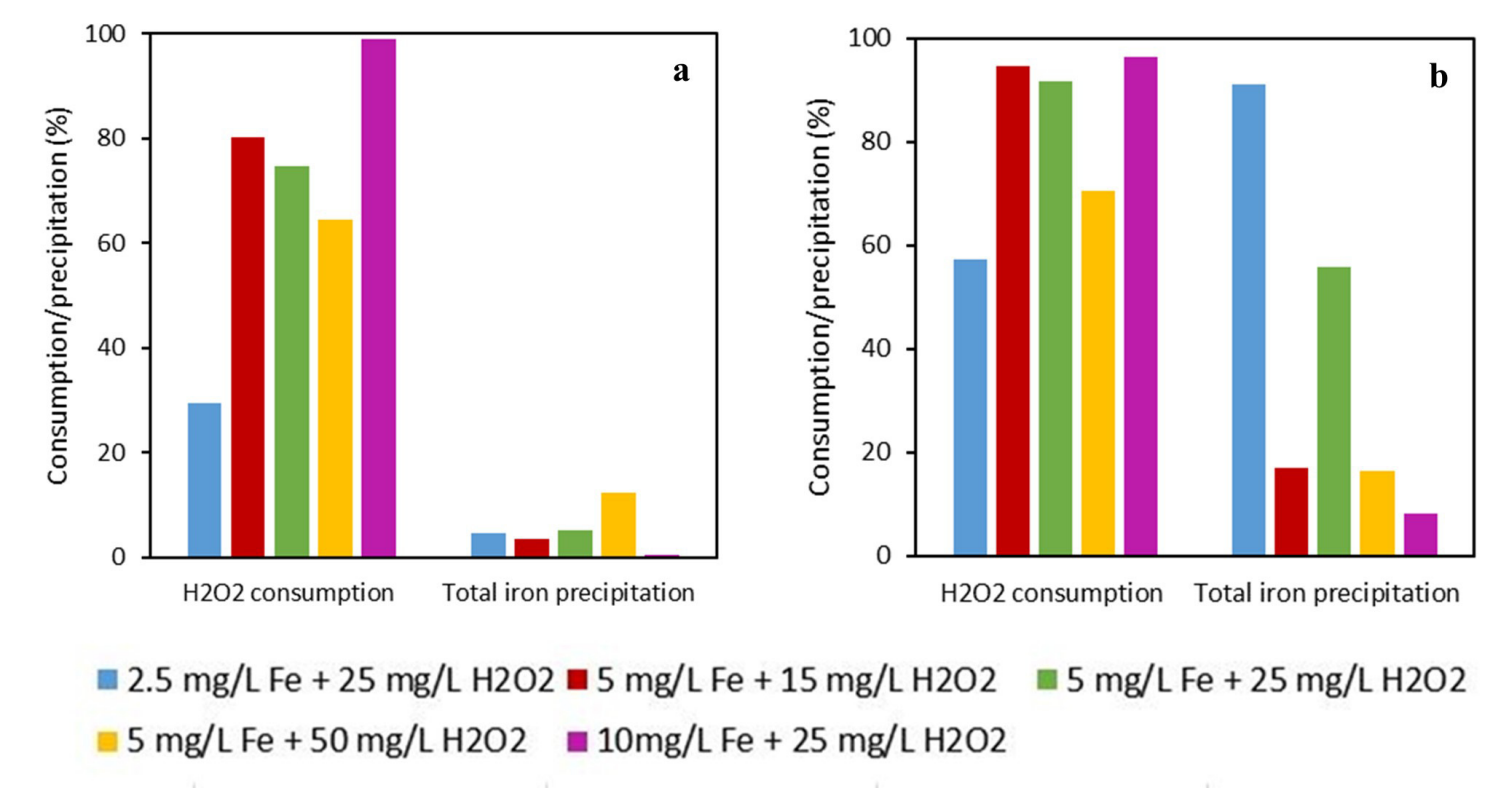
H _2_O _2_ consumption and total iron precipitation in the experiments performed with **a**) DTPA-Fe **b**) EDDS-Fe in photo-Fenton process with different initial concentrations of Fe (III) and H _2_O _2_ [IMID] _0_=[DIU] _0_=[PROPI] _0_= 1 mg L ^-1^.

From the results displayed in Figure 2– Figure 5, it can be observed that the experiments carried out with EDDS, the observed kinetics at initial times (k1) were always higher than the experiments using DTPA. For instance, IMID was degraded at about 80% in only 15 minutes with EDDS using 2.5 mg L ^-1^ of Fe and 25 mg L ^-1^ of H _2_O _2_; however, while employing the same conditions, less than 30% was degraded using DTPA. The same results were obtained in diverse studies performed by López-Vinent and colleagues ( López-Vinent *et al.*, 2020; López-Vinent *et al.*, 2021a; López-Vinent *et al.*, 2021b). These differences could be related to the consumption of H _2_O _2_, which always was higher in the experiments performed with EDDS than DTPA. The results are displayed in Figure 6.

The high H _2_O _2_ consumption leads to the high production of hydroxyl radicals and subsequent oxidation of MPs. This fact is also related to the stability of chelating agents with iron (III). DTPA presents a higher stability constant with iron than EDDS. Lower stability allows a high reaction between iron and H _2_O _2_, increasing the hydroxyl radical production. However, because of that, the iron precipitation was higher in the experiments performed with EDDS-Fe. The low stability of EDDS with iron allowed high MP removal rates in a few minutes and it diminished the requirement of high doses of iron and H _2_O _2_. Nevertheless, the higher iron precipitation with EDDS than DTPA caused the formation of oxohydroxides, which are not soluble and less photoactive than dissolved iron. This decreased the efficiency of the treatment, leading to an almost-plateau curve from the 15-minute mark until the end of the treatment, not reaching total MP removal overall in IMID and PROP.

Finally, concerning the differences in the removal kinetic in the first 15 min of the experiments performed with EDDS-Fe and DTPA-Fe, a previous study ( López-Vinent *et al.*, 2022) revealed the role of sunlight in the photoexcitation of iron complexes to generate additional ROS without H _2_O _2_ in the treatment. Further studies should be performed to confirm the extension of this contribution on the MP removal kinetics observed by DTPA-Fe and EDDS-Fe.

### Influence of components contained in pineapple washing wastewater

As observed in the previous section, iron complexes present different efficiencies in MP removal, which islinked to the constant stability of each chelating agent with iron. It was revealed that EDDS-Fe presented higher kinetics in MPs degradation than DTPA-Fe, but also showed higher iron precipitation. This fact decreases the efficiency of the process due to the low photocatalytic activity. Additionally, its effect could be more noticeable in a complex matrix. For instance, López-Vinent and coworkers ( López-Vinent *et al.*, 2021b) investigated the effect of the matrix on iron precipitation and subsequent decrease in MP removal efficiency. In that work it was revealed that the iron precipitation was 1.8 times higher in a complex matrix than another one with low dissolved organic carbon (DOC) and alkalinity. Additionally, the organic matter and alkalinity present in the matrix also compete for hydroxyl radicals, decreasing the efficiency of the MP removal process.

**Figure 7.**
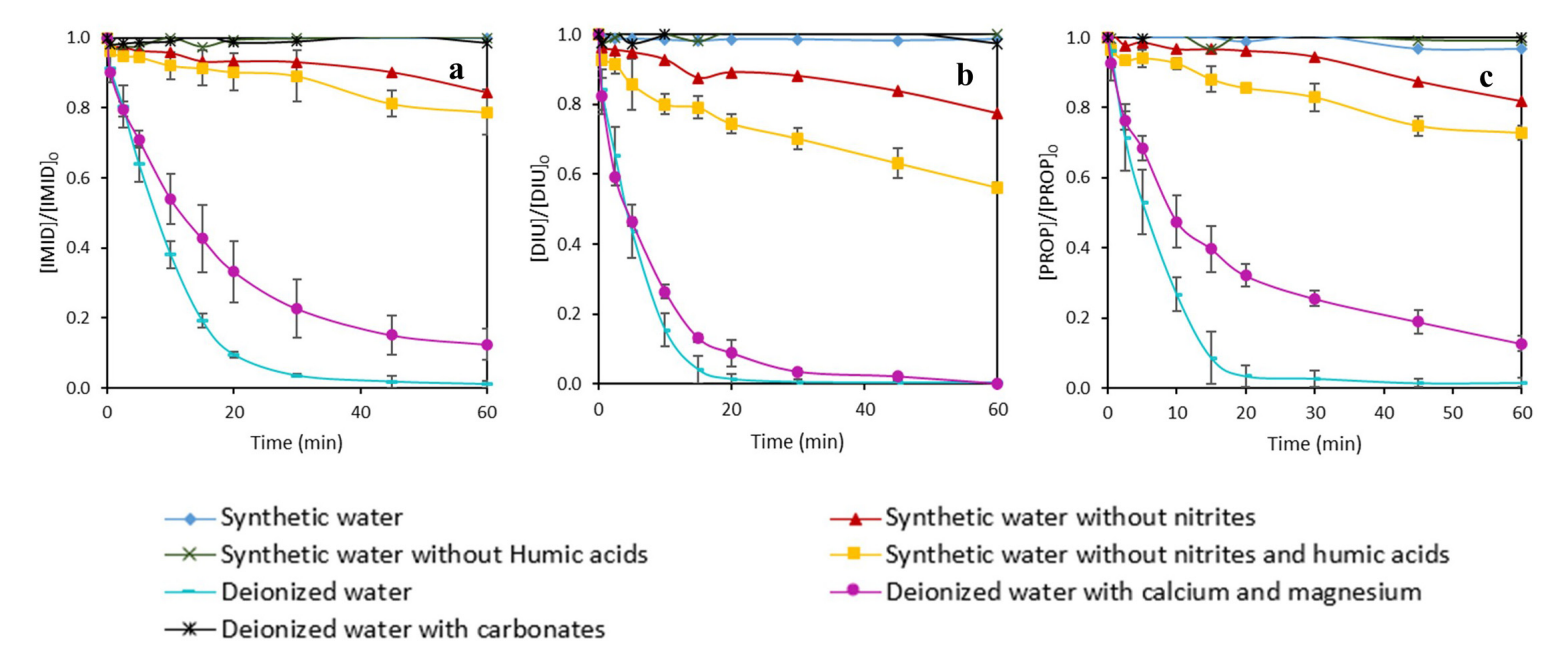
Influence of the different components contained in pineapple processing wastewater in the simultaneous removal of **a**) imidacloprid (IMID), **b**) diuron (DIU) and **c**) propiconazole (PROP) by photo-Fenton process using DTPA as an iron chelate. [IMID] _0_=[DIU] _0_=[PROPI] _0_= 1 mg L ^-1^; [Fe(III)]= 10 mg L ^-1^; [H _2_O _2_]= 25 mg L ^-1^.

**Figure 8.**
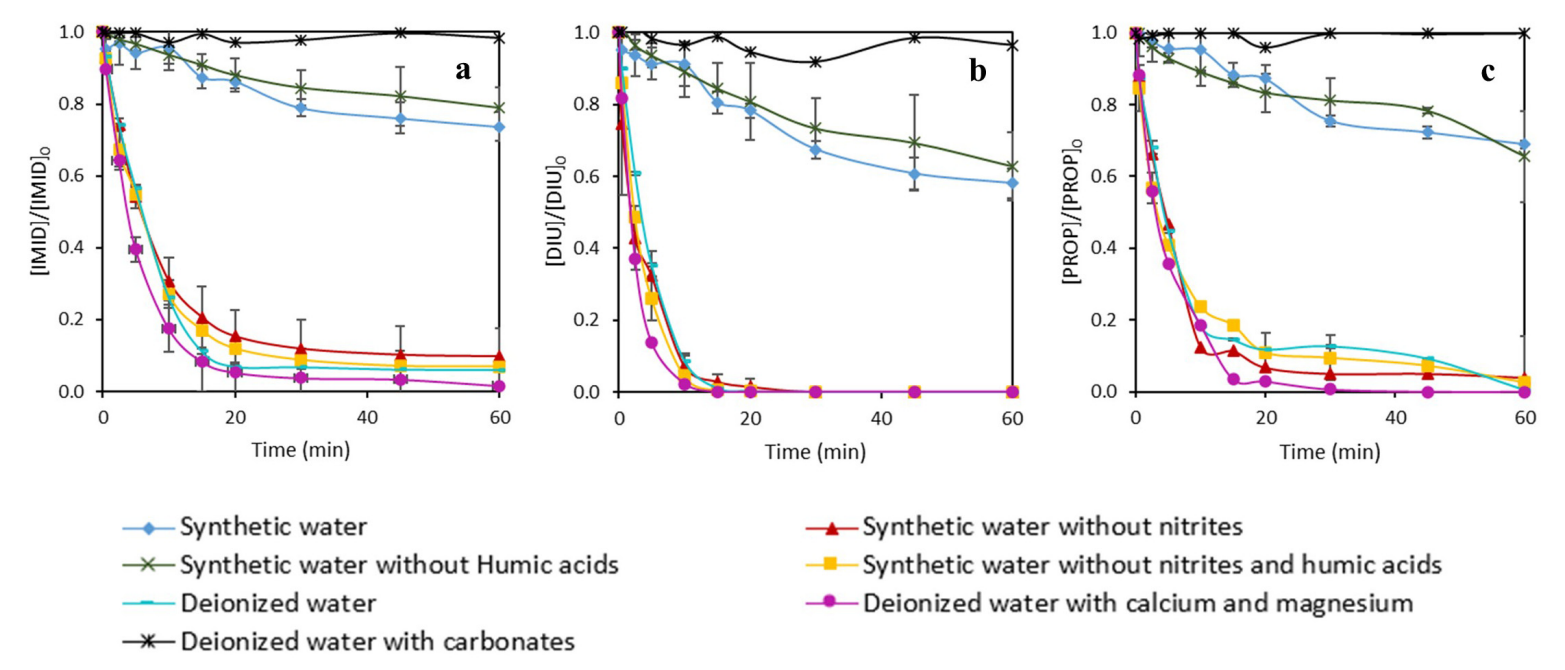
Influence of the different components contained in pineapple processing wastewater in the simultaneous removal of **a**) imidacloprid (IMID), **b**) diuron (DIU) and **c**) propiconazole (PROP) by photo-Fenton process using EDDS as an iron chelate. [IMID] _0_=[DIU] _0_=[PROPI] _0_= 1 mg L ^-1^; [Fe(III)]= 10 mg L ^-1^; [H _2_O _2_]= 25 mg L ^-1^.

**Figure 9.**
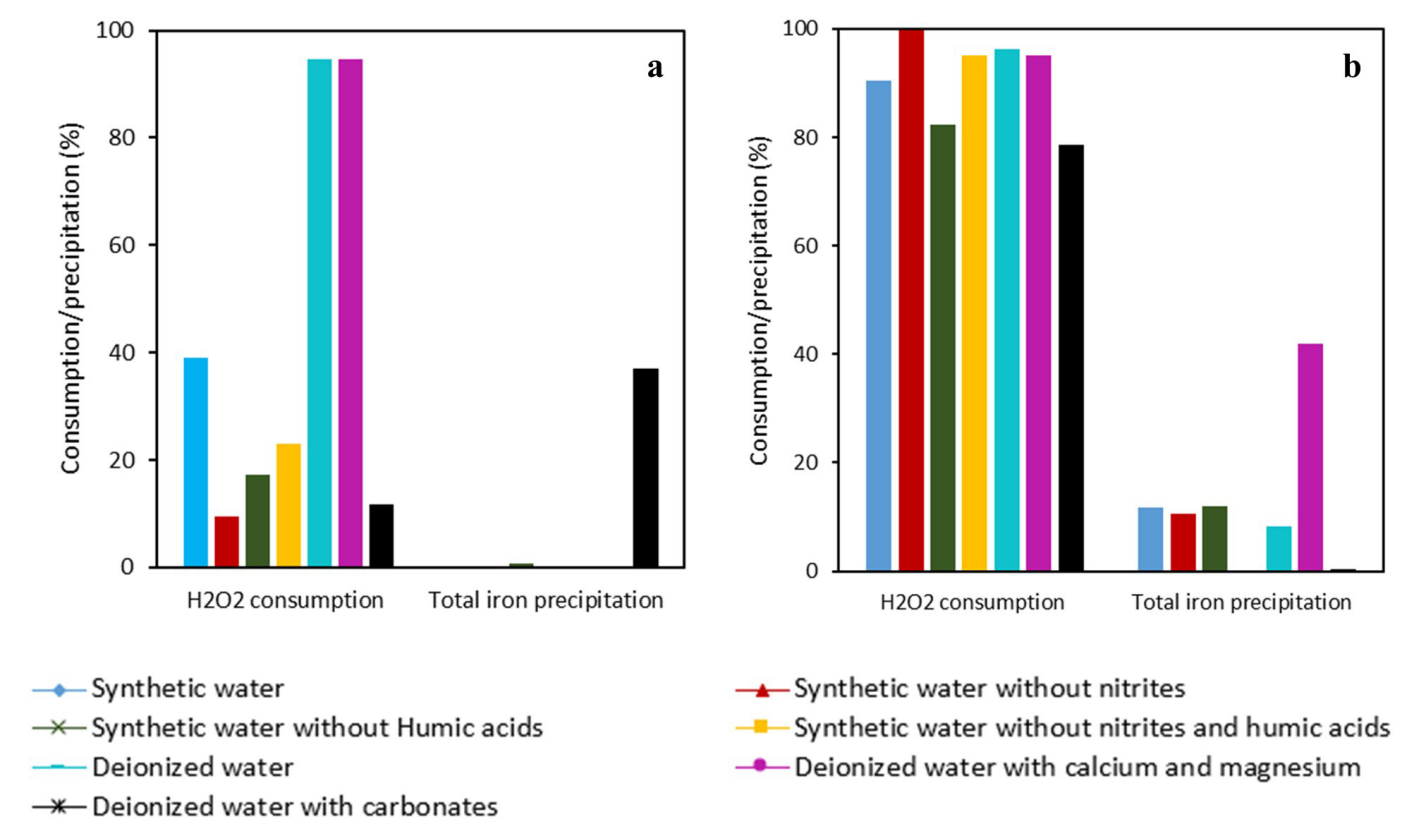
H _2_O _2_ consumption and total iron precipitation in the experiments performed with **a**) DTPA-Fe **b**) EDDS-Fe in photo-Fenton process with synthetic pineapple processing wastewater. [IMID] _0_=[DIU] _0_=[PROPI] _0_= 1 mg L ^-1^; [Fe(III)]= 10 mg L ^-1^; [H _2_O _2_]= 25 mg L ^-1^.

For these reasons, in this work, experiments with synthetic wastewater simulating the pineapple processing wastewater were carried out with both iron complexes, EDDS-Fe and DTPA-Fe using 10 mg L ^-1^ of Fe(III) and 25 mg L ^-1^ of H _2_O _2_. The properties of synthetic wastewater were reported in the Methods section. Moreover, experiments testing components separately in deionized water or removing some components of synthetic water, were also performed in order to investigate the effect of each component on the MPs efficiency decrease. The results of MPs removal are displayed in Figure 7 and Figure 8, for DTPA-Fe and EDDS-Fe, respectively. Additionally, total iron precipitation and H _2_O _2_ consumption are shown in Figure 9.

The removal rates found in synthetic water were <5% with DTPA and between 25-45% with EDDS, and both were much lower than with deionized water (>95% for both chelates) for the three MPs ( Figure 7 and Figure 8), evidencing the hindering effect of a complex matrix (synthetic water) and the different effect of both chelates on the treatment efficiency. Regarding the experiments removing some of the constituents of synthetic water, the highest removal rates were achieved with deionized water with Ca ^2+^ and Mg ^2+^ using EDDS (more than 90% in only 30 min). These removal rates were similar to deionized water, which indicates that Ca ^2+^ and Mg ^2+^ did not significantly hinder the treatment efficiency of the photo-Fenton process using EDDS. The experiments performed with DTPA were quite different. Although the tests carried out in deionized water with Ca ^2+^ and Mg ^2+^ achieved the best removal rates compared to other experiments, the degradations were not equal to tests without any constituent (in deionized water). For instance, in 20 min, only 65% of PROP was removed in the presence of Ca ^2+^ and Mg ^2+,^ while more than 90% removal was observed in deionized water.

Removing nitrites from the synthetic water improved the efficiency of the treatment with both chelates, although at lower rates for DTPA (removal rates were 20–25% and >90% for DTPA and EDDS, respectively, compared to <5% for DTPA and 25–45% for EDDS both with synthetic water). Thus, the efficiency of the treatment when removing nitrites was improved further to 20–45% for DTPA, maintaining high rates (>90%) for EDDS. However, removing only humic acids from the synthetic water did not seem to affect the efficiency of the treatment (removal rates without humic acids were <5% and 20-40% for DTPA and EDDS, respectively). These results indicate that nitrites had a hindering effect on the efficiency of the treatment which is potentiated in the presence of humic acids.

With regards to the different effects of both chelates, we found that removal of MPs was higher with EDDS than with DTPA ( Figure 7 and Figure 8). This difference can be related to the higher consumption of H _2_O _2_ with EDDS than DTPA ( Figure 9) which has been previously discussed ( *Determination of effective H2O2 and Fe(III) concentrations* section). Briefly, the lower stability of EDDS with iron enables a faster/greater production of hydroxyl radicals, increasing removal rates for the MPs. This would have allowed overcoming the hindering effect observed for some of the synthetic water constituents with DTPA ( *i.e.* synthetic water, nitrites and nitrites potentiated with humic acids, see previous paragraphs). However, we also found that iron was still dissolved and H _2_O _2_ has not been completely consumed at the end of the experiments with DTPA ( Figure 9). Since MPs exhibited declining trends until the end of the experiments with DTPA, it is likely that the photo-Fenton reaction would have continued after the experiment. This has been observed by Lopez Vinent *et al.* (2021a), who found that declining of propranolol hydrochloride, acetamiprid and sulfamethoxazole were slower with DTPA than with EDDS, but the dissolved iron and remaining H _2_O _2_ with DTPA allowed the reaction to continue until reaching same/higher removal rates than EDDS. Further experiments are required to confirm whether the trends we found with DTPA would allow the reaction to continue.

We also found that the removal rates with deionized water with only carbonates, at a higher concentration (150 mg L ^-1^) than in synthetic water, were between 0 and 4% for both chelates, being even lower than with synthetic water. Such low removal rates may be associated to the alkaline pH ( *i.e.* we found pH values >10) resulting from dissolving carbonates, but also to the carbonate anions themselves. High pH values could have produced the dissociation of Fe (III) from the chelates, which is in line with the total iron precipitation we found with DTPA for water with carbonates ( Figure 9). Carbonate anions have been shown to interact with iron, generating less reactive species and severely limiting the Fenton process ( Vallés *et al.*, 2021). Whichever the mechanism, the effect from carbonates at low concentration (30 mg L ^-1^) seemed to disappear in presence of the other constituents of synthetic water using EDDS. But in experiments carried out with DTPA, the negative effect was also observed even at a low concentration of carbonates, since in the tests performed in synthetic water without nitrites and carbonates, the removal rates were lower than experiments in deionized water. Thus, based on our results, nitrites should be removed from pineapple water before the photo-Fenton process using EDDS in order to efficiently reduce the studied MPs.

**Figure 10.**
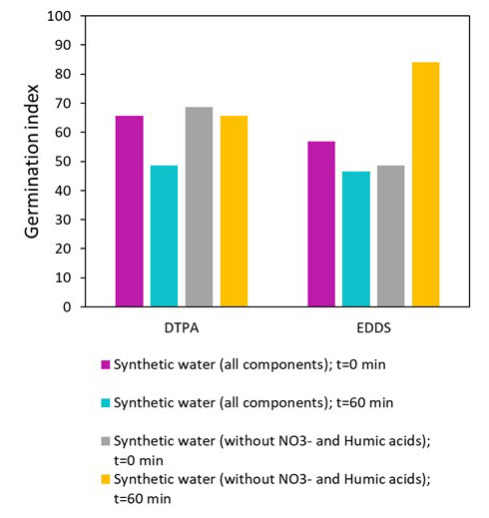
Phytotoxicity evaluation in effluents at initial and at the end of the photo-Fenton treatment using DTPA and EDDS in synthetic water and synthetic water without nitrites and humic acids.

Finally, phytotoxicity using seeds of *E. sativa* was also evaluated in the experiments performed with synthetic water and synthetic water without nitrites and humic acids, to assess the suitability of the effluent to be reused in irrigation. The results are displayed in Figure 10.

As can be observed in Figure 10, the germination index (GI) before the treatment (t=0) was about 65-70% in the samples with DTPA. Using EDDS, the germination index was quite lower (about 50%). However, no significant injury to the plant was observed according to Zucconi and coworkers ( Zucconi *et al.*, 1981a; Zucconi *et al.*, 1981b) in both cases. Zucconi *et al.* proposed different phytotoxicity categories depending on the inhibition percentage of the GI

- Inhibition of seed germination and root elongation: <20% GI.
- Presence of phytotoxicity: 20–50% GI.
- No significant injury to the plant: >50–60% GI.
- Disappearance of phytotoxicity: >80–85% GI.
- Stimulation of the root elongation: >100% GI (better than control, which represents 100% of GI)).

Regarding the experiments at the end of the treatment, clear differences were observed between the effluents tested. With synthetic water in both cases, using EDDS or DTPA, the GI decreased compared to the initial time. In that case, the GI dropped by 17% and 10% for DTPA and EDDS, respectively. Nevertheless, that fact was not unexpected, since no removal of MPs was observed using DTPA, or low rates (about 30% removal for each MP) were obtained using EDDS. This fact could be linked to the presence of by-products ( Freitas *et al.*, 2017) probably more toxic than the initial MPs, as well as to the transformation products from the iron complexes. The same behavior was observed in the study of López-Vinent and colleagues ( López-Vinent *et al.*, 2021b). Concerning the phytotoxicity in synthetic water without nitrites and humic acids, it was observed that the same germination index was obtained at the start and end of the treatment using DTPA (about 70%) which does not imply significant injury to the plant. That fact, in part, could be related to the formation of toxic by-products which decrease the germination index, but could also be linked to the disappearance of the diuron, which is a herbicide. Finally, the results on phytotoxicity in the experiments using EDDS revealed an increase of the germination index at the end of the treatment compared to the initial time, achieving a germination index value of 84%. This value represents a disappearance of the phytotoxicity, according to Zucconi and coworkers. The results are in accordance with the removals of MPs, since at the end of the treatment more than 90% were removed and total diuron degradation was observed in only 20 min. The higher kinetic rates using EDDS than DTPA could imply the removal of the possible by-products formed during the treatment, which results in low phytotoxicity to the seeds. So, this means that the effluent treated with EDDS-Fe would be suitable for reuse in agricultural irrigation without causing any damage to crops. However, it needs more investigation since continued irrigation with traces of diuron, propiconazole or imidacloprid could create resistance to these compounds in crops.

## Conclusions

This work demonstrated the suitability of photo-Fenton process at natural pH to remove a mixture of imidacloprid, diuron and propiconazole. The operational conditions were optimized for both iron complexes and the results revealed that high kinetic rates on MP removal were obtained using 10 mg L ^-1^ of Fe(III) and 25 mg L ^-1^ of H _2_O _2_. With these conditions, more than 80% of three micropollutants were removed in only 20 min for both iron complexes. In all cases, experiments using EDDS-Fe achieved higher kinetic rates than DTPA-Fe. These differences could be related to the stability constant of each chelating agent with iron. EDDS presents a lower constant than DTPA, resulting in a high availability of H _2_O _2_ and light to react with iron. The differences between them increased in the tests with the lowest iron concentration (2.5 mg L ^-1^ of Fe (III)), revealing that iron concentration is a key parameter on the removal kinetics. In the case of EDDS-Fe the differences between different iron and H _2_O _2_concentration were lower than using DTPA-Fe, which also could be linked to the stability constant of iron complexes.

The efficiency of the photo-Fenton process using synthetic pineapple processing wastewater was also investigated in this work. In this water, the removal of three micropollutants was decreased for both iron complexes compared to the removals obtained using deionized water. No micropollutant removal was achieved using DTPA-Fe and a low degradation was achieved with EDDS-Fe (for instance, only 40% removal of diuron was achieved at 60 min). In this sense, the effect of each component on MP degradation was investigated. The effect of the compounds was different depending on the iron complex used. Nitrites presented the most influence on EDDS-Fe, while carbonates and humic acids did not affect the MPs degradation. However, in the case of tests performed with DTPA-Fe, a four-time decrease was observed when the water contains carbonates and humic acids compared to the results in deionized water. In that case, the nitrites did not significantly influence MP removal. On the other hand, the concentration of calcium and magnesium also only affected the test using DTPA-Fe. But in that case, the effect was lower since differences on MP removal rates lower than 20% were observed at the end of the process compared to tests in deionized water.

Finally, phytotoxicity was investigated in the tests performed with synthetic water and synthetic water without nitrites and humic acids. The results revealed that with synthetic water, phytotoxicity was observed (less than 50% of germination index) at the end of the treatment with both iron complexes. However, in the tests carried out in synthetic water without nitrites and humic acids, no significant injury to the plant and disappearance of phytotoxicity were observed in the tests with DTPA-Fe and EDDS-Fe, respectively. These results disclosed the suitability of treated effluent with EDDS-Fe for reuse in the agricultural sector when the content of nitrites does not compromise MP removal. 

## Data availability

### Underlying data

Dataversecat: Experimental results obtained by the application of solar-based photo-Fenton process at natural pH for pineapple processing wastewater treatment. https://doi.org/10.34810/data210 ( Bolaños *et al.*, 2022)

This project contains the following underlying data in KNOWPEC-FINAL.xlsx:

- Sheet 1: Control test. Photo-Fenton test without chelates and photolysis tests
- Sheet 2: Experiments of removal of imidachloprid, diuron and propiconazole, by solar photo-Fenton with the chelate DTPA-Fe (III), per triplicate, at the following conditions:
  - 2.5 mg/L Fe + 25 mg/L H _2_O _2_
  - 5 mg/L Fe + 15 mg/L H _2_O _2_
  - 5 mg/L Fe + 25 mg/L H _2_O _2_
  - 5 mg/L Fe + 50 mg/L H _2_O _2_
  - 10 mg/L Fe + 25 mg/L H _2_O _2_
- Sheet 3: Obtention of the first-order kinetic constants of imidachloprid, diuron and propiconazole removal from experiments in Sheet 2.
- Sheet 4: Figures of average data of experiments with the chelate DTPA-Fe (III), presented in sheet 2.
- Sheet 5: Experiments of removal of imidachloprid, diuron and propiconazole, by solar photo-Fenton with the chelate EDDS-Fe (III), per triplicate, at the following conditions:
  - 2.5 mg/L Fe + 25 mg/L H _2_O _2_
  - 5 mg/L Fe + 15 mg/L H _2_O _2_
  - 5 mg/L Fe + 25 mg/L H _2_O _2_
  - 5 mg/L Fe + 50 mg/L H _2_O _2_
  - 10 mg/L Fe + 25 mg/L H _2_O _2_
- Sheet 6: Obtention of the first-order kinetic constants of imidachloprid, diuron and propiconazole removal from experiments in Sheet 5.
- Sheet 7: Figures of average data of experiments with the chelate EDDS-Fe (III), presented in sheet 2.
- Sheet 8: Experiments of removal of imidachloprid, diuron and propiconazole, by solar photo-Fenton with the chelate DTPA-Fe (III), per triplicate, at 10 mg/L Fe + 25 mg/L H _2_O _2_, in synthetic water (composition as indicated in the sheet nº 8)
- Sheet 9: Experiments of removal of imidachloprid, diuron and propiconazole, by solar photo-Fenton with the chelate EDDS-Fe (III), per triplicate, at 10 mg/L Fe + 25 mg/L H _2_O _2_, in synthetic water (composition as indicated in the sheet nº 9)
- Sheet 10: Results of phytotoxicity of samples treated with the chelates DTPA-Fe (III) and EDDS-Fe (III) and at 10 mg/L Fe + 25 mg/L H _2_O _2_, in synthetic water (composition as indicated in the sheet nº 10)

Data are available under the terms of the Creative Commons Attribution 4.0 International license (CC-BY 4.0).
