# Supplementary material for: Use of organic fertilizers in solar photo-Fenton process as potential technology to remove pineapple processing wastewater in Costa Rica
Source: Open Res Eur. 2022 Sep 1;2:105. [Version 1] doi: 10.12688/openreseurope.14997.1 (PMC10445884; doi:10.12688/openreseurope.14997.1)
Supplement: Peer review history

# Peer review: Reviewer response for version 1

**DOI:** 10.21956/openreseurope.16212.r34172

**Published:** 2023-08-31

**Authors:** Marwa El-Azazy

**Affiliations:** 1 Qatar University, Doha, Doha, Qatar

The current article is addressing the removal of three micropollutants (three pesticides) from pineapple processing water using two organic fertilizers; DTPA-Fe and EDDS-Fe as iron chelates. Authors have reported the implementation of the two chelates at near neutral pH for solar-driven photo-Fenton process. Reported findings showed the efficiency of the mentioned approach in pure water with % removal exceeding 80% in a timeframe of 20 min. Yet, interferents diminished the applicability of the proposed approach to synthetic samples. To that end, the article in general is well written, the discussion of the findings is well displayed, and the proposed approach is applicable on the lab scale. Yet, and to improve the quality of the paper, authors need to address the following comments:

1. The title is long and could be shortened. The current title might not be reflecting the removal of the pesticides from the pineapple processing water.
2. It would be better (and to show the magnitude of the problem) if authors could indicate the concentration levels at which the 28 pesticides were found.
3. Please check the DT50 of Imidacloprid.
4. Please show the chemical structure of the three pesticides as well as the chelating agents.
5. Please consider using more recent references in the introduction section.
6. Authors need to add a table after the introduction section that shows the reported methods for the removal of the three MPs, the reported % removal, what kind of water sample was investigated and the removal conditions (pH, concentrations, etc.)
7. Iron chloride (FeCl _3_ ·6H _2_O) should be Iron (III) chloride hexahydrate.
8. The sentence “To prepare the dissolution with the iron chelate DTPA-Fe, an appropriate amount of it was added to Milli-Q water” needs clarification. Authors need to either state the ‘appropriate amount’ or rephrase the sentence and clarify the meaning of the sentence ‘To prepare the dissolution’.
9. Similarly, the next sentence ‘The concentrations were calculated according to the percentage of iron content (7%) in order to obtain concentrations of 2.5, 5 and 10 mg L ^-1^ of iron in solution’. Authors already mentioned that the starting concentration of DTPA-Fe is 7% under the experimental section so no need for repetition to avoid confusion.
10. In the sentence ‘In the mixture with EDDS, which was not acquired as iron chelate, the same molar ratio than DTPA: Fe was selected’, I think authors meant ‘like’ rather than ‘than’.
11. Better to use ‘withdrawn, drawn, taken out, removed’ instead of ‘‘retired’ periodically from the reactor during 60 min’.
12. In the same context, please clarify the time points at which you have withdrawn the samples over the 60 min – e.g., 0, 30 s, 1, 2, … min.
13. Authors need to justify the use of different concentrations of iron and hydrogen peroxide and the ratio of iron: hydrogen peroxide.
14. A justification of the different degradation percentages observed among the three MPs based on their chemical structures is needed and would help understanding the degradation mechanism.
15. Please be consistent in using abbreviations throughout the manuscript text (e.g., minutes and min., K1 and K _1_, etc.).
16. As shown by the findings in synthetic wastewater, the interferents hindered or diminished the removal of the MPs, an issue which limits the applicability of the proposed approach on a large scale and on real samples. Therefore, authors need to find an alternative approach or work on adjusting the experimental conditions, especially the pH to fit the large-scale application.

Is the study design appropriate and does the work have academic merit?

Yes

Is the work clearly and accurately presented and does it cite the current literature?

Partly

If applicable, is the statistical analysis and its interpretation appropriate?

Not applicable

Are all the source data underlying the results available to ensure full reproducibility?

Yes

Are the conclusions drawn adequately supported by the results?

Yes

Are sufficient details of methods and analysis provided to allow replication by others?

Yes

Reviewer Expertise:

Wastewater treatment, recycling of agro-wastes into value-added products, experimental design

I confirm that I have read this submission and believe that I have an appropriate level of expertise to confirm that it is of an acceptable scientific standard, however I have significant reservations, as outlined above.

# Peer review: Reviewer response for version 1

**DOI:** 10.21956/openreseurope.16212.r34170

**Published:** 2023-08-18

**Authors:** Antonino Fiorentino

**Affiliations:** 1 University of Salerno, Fisciano, Italy

In my opinion, the work displays a well-organized structure and features an intriguing comparison between two organic binders utilized in solar Fenton processes. The paper's strength lies in the comprehensive comparison between deionized water and wastewater, as it takes into account numerous variables that exert an influence on the Fenton process. Below, I offer specific suggestions aimed at assisting the authors in enhancing the quality of their paper:

1. I recommend that the authors revise the first sentence of the abstract to enhance its clarity. Proposed revision: "This study explores the application of organic fertilizers, specifically DTPA-Fe and EDDS-Fe, as iron chelates within a solar-driven photo-Fenton process conducted at natural pH."
2. Within the introduction, it seems appropriate to replace the term "decreet" with "decree."
3. To ensure verbal consistency, consider modifying the phrase "imidacloprid was also detected in groundwater" to "imidacloprid has also been identified in groundwater" in the introduction.
4. In the introduction, the phrase "DT50 of 174–91" might be more suitable as "DT50 of 174–191."
5. Within the experimental procedures, kindly specify the distance maintained between the lamp and the sample.
6. In the results section, make sure to include the definitive article before the abbreviation of pollutants, which appears to have been omitted in several instances.
7. The caption for Figure 9 requires adjustment. While the colors correspond to lines, they should align with the histograms.
8. A sentence on page 11 appears somewhat unclear: "Thus, the efficiency of the treatment when removing nitrites was improved further to 20–45% for DTPA, maintaining high rates (>90%) for EDDS." Consider revising this sentence for greater clarity.
9. I propose that the authors incorporate a discussion concerning the rationale behind their choice of Iron and Hydrogen Peroxide concentrations

Is the study design appropriate and does the work have academic merit?

Yes

Is the work clearly and accurately presented and does it cite the current literature?

Yes

If applicable, is the statistical analysis and its interpretation appropriate?

Yes

Are all the source data underlying the results available to ensure full reproducibility?

Yes

Are the conclusions drawn adequately supported by the results?

Yes

Are sufficient details of methods and analysis provided to allow replication by others?

Yes

Reviewer Expertise:

Treatment of civil and industrial wastewater through advanced oxidation and adsorption processes. Drinking water treatment. Removal of emerging contaminants. Environmental impact assessment.

I confirm that I have read this submission and believe that I have an appropriate level of expertise to confirm that it is of an acceptable scientific standard.

# Peer review: Reviewer response for version 1

**DOI:** 10.21956/openreseurope.16212.r30042

**Published:** 2022-09-27

**Authors:** Irene Salmeron Garcia

**Affiliations:** 1 Chair of Urban Water Treatment, University of Luxembourg, Esch-sur-Alzette, Luxembourg

This manuscript assesses the feasibility of photo-Fenton treatment as an alternative for the removal of three organic pesticides commonly contained in an agro-industrial wastewater. Two different iron sources were studied for the operation at natural pH as well as the influence of the crucial ions and substances present on the matrix.

 The work is well structured, well explained and results and discussion are coherent and in line with other works previously reported in literature. However, some minor comments arise and would need to be addressed for the final version of the document. The conclusions seem more a summary of the work done than the main findings obtained from the study. In fact, in conclusions it is stated that photo-Fenton is suitable for the removal of the three MP at near-neutral pH, but this only happened in pure water and not in the case of the synthetic agro-industrial effluent, where only a small ratio of removal was achieved, so probably for that kind of effluents would be better the application of an alternative technology.

 Regarding the text, it is reported that the removal kinetics are linked to the stability of the chelating agent with iron. This argument is repeated several times along the text and for an easier understanding and a more friendly reading I would avoid that redundancy.

 The implementation of these minor changes would improve the quality of the document.

Is the study design appropriate and does the work have academic merit?

Yes

Is the work clearly and accurately presented and does it cite the current literature?

Yes

If applicable, is the statistical analysis and its interpretation appropriate?

Not applicable

Are all the source data underlying the results available to ensure full reproducibility?

Yes

Are the conclusions drawn adequately supported by the results?

Partly

Are sufficient details of methods and analysis provided to allow replication by others?

Yes

Reviewer Expertise:

Micropollutants removal from wastewater treatment effluents

I confirm that I have read this submission and believe that I have an appropriate level of expertise to confirm that it is of an acceptable scientific standard.
